# Application of Purified Botulinum Type A Neurotoxin to Treat Experimental Trigeminal Neuropathy in Rats and Patients with Urinary Incontinence and Prostatic Hyperplasia

**DOI:** 10.1155/2012/648384

**Published:** 2012-06-14

**Authors:** Yoshizo Matsuka, Teruhiko Yokoyama, Yumiko Yamamoto, Tomonori Suzuki, Ni Nengah Dwi Fatmawati, Atsushi Nishikawa, Tohru Ohyama, Toshihiro Watanabe, Takuo Kuboki, Atsushi Nagai, Keiji Oguma

**Affiliations:** ^1^Department of Oral Rehabilitation and Regenerative Medicine, Okayama University Graduate School of Medicine, Dentistry and Pharmaceutical Sciences, Okayama 700-8558, Japan; ^2^Department of Urology, Kawasaki Medical School, Kurashiki 701-0192, Japan; ^3^Department of Medical Bacteriology, Okayama University Graduate School of Medicine, Dentistry and Pharmaceutical Sciences, 2-5-1 Shikata-cho, Okayama 700-8558, Japan; ^4^Department of Applied Biological Science, Tokyo University of Agriculture and Technology, Tokyo 183-8509, Japan; ^5^Department of Food and Cosmetic Science, Faculty of Bioindustry, Tokyo University of Agriculture, Abashiri 099-2493, Japan

## Abstract

Type A neurotoxin (NTX) of *Clostridium botulinum* was purified by a simple procedure using a lactose gel column. The toxicity of this purified toxin preparation was retained for at least 1 year at −30°C by supplementation with either 0.1% albumin or 0.05% albumin plus 1% trehalose. When purified NTX was used to treat 49 patients with urinary incontinence caused by either refractory idiopathic or neurogenic detrusor overactivity, 36 patients showed significant improvement in symptoms. These beneficial effects were also observed in cases of prostatic hyperplasia. The results obtained with NTX were similar to that of Botox. The effects of NTX on trigeminal neuralgia induced by infraorbital nerve constriction (IoNC) in rats were also studied. Trigeminal ganglion neurons from ipsilateral to IoNC exhibited significantly faster onset of FM4-64 release than sham-operated contralateral neurons. Intradermal injection of NTX in the area of IoNC alleviated IoNC-induced pain behavior and reduced the exaggerated FM4-64 release in trigeminal ganglion neurons.

## 1. Introduction

 In this manuscript, three main topics are described: (1) the structure of type A progenitor toxin and purification of type A neurotoxin by a lactose gel column based on progenitor toxin characteristics; (2) treatment of patients with urinary incontinence and prostate hyperplasia with this purified neurotoxin; (3) application of the purified neurotoxin for treating trigeminal neuralgia in a rat model. Introduction including the purpose of these three topics is described at the beginning of each paragraph. 

## 2. Structure of Type A Progenitor Toxin and Purification of Type A Neurotoxin

*Clostridium botulinum* strains produce immunologically distinct neurotoxins (NTX; types A to G) with molecular masses (M_r_) of approximately 150 kDa. In an activated form, the NTXs have a cleavage site at the one third position of their N terminus conjugated with an S–S bond, which can be cleaved by cellular proteases or exogenously added trypsin. In culture fluid and in acidic foods, the NTXs associate with nontoxic components and form large complexes designated progenitor toxins (PTXs). PTXs are found in three forms with M_r_ of 900 kDa (19S, LL), 500 kDa (16S, L), and 300 kDa (12S, M); later, it became clear that the actual M_r_ of the L and LL toxins is much larger than 500 and 900 kDa, respectively (see below). The M toxin is composed of an NTX and a nontoxic component having no hemagglutinin (HA) activity (designated nontoxic non-HA; NTNH), whereas the L and LL toxins are composed of an NTX(s), an NTNH(s), and HAs. The type A strain produces three forms of toxin (M, L, and LL), while types B, C, and D produce M and L toxins. In alkaline conditions in the absence of gastric juices, the PTXs dissociate into the NTX(s) and nontoxic components, L and LL toxins (designated HA-positive PTXs) dissociate into an NTX(s), and a nontoxic component (complex) of an NTNH(s) and HAs, and the M toxin dissociates into an NTX and an NTNH [1].

**Figure 1:**
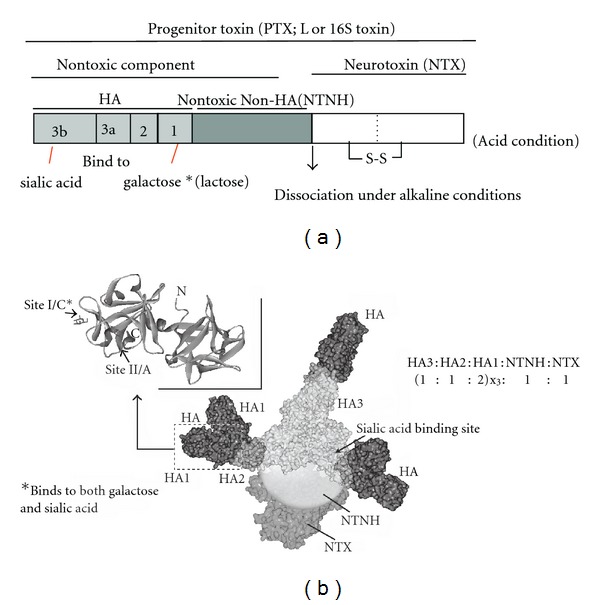
Progenitor L toxin structure. (a) Scheme indicating that (1) under alkaline conditions PTXs dissociate into an NTX and nontoxic components; (2) fully activated NTX is cleaved at a site in the N-terminus; (3) HA1 and HA3b bind galactose and sialic acid, respectively. (b) Predicted tertiary structure of type C and D L toxins and molar ratio of their (sub)components. Model is based on the structural data of Hasegawa et al. [8] and Nakamura et al. [7]. Three dimensional structure of HA1 and its binding sites for sugars are also shown. Both type A and C HA1 showed similar structures consisting of two *β*-trefoil domains conjugated by an *α*-helix. Two sugar-binding sites (I and II) on C-terminal *β*-trefoil domain are predicted and A-HA1 and C-HA1 binds to galactose via sites II and I, respectively. In the case of type C, HA1 also bound to sialic acid via site I [6].

 We found that (1) LL toxin is a dimer of L toxins; (2) cleavage or deletion is detected in the N-terminal region of the M toxin NTNH; (3) HA consists of three to four subcomponents designated HA-1, -2, and -3 (-3a, -3b); (4) HA1 and HA3b bind to galactose and sialic acid, respectively, present on the surface of erythrocytes and small intestine epithelial cells [2–4]. Recently, we also analyzed the crystal structures of type A and C HA1, and type C HA3 to describe their sugar binding sites [5–7]. Furthermore, by investigating the tertiary structure of type C and D L toxins with electron microscopy, the molar ratio of NTX : NTNH: (HA1 : HA2 : HA3) was predicted to be 1 : 1 : (2 : 1 : 1)_x3_, and the actual L toxin M_r_ was calculated to be approximately 750 kDa. The LL toxin M_r_ was, therefore, estimated to be about 1,500 kDa because it is a dimer of the L toxin, although its tertiary structure remains unclear [7, 8]. Some of these data are summarized in Figure 1.

Type A and B PTXs have been used to treat patients with strabismus, blepharospasm, nystagmus, facial spasm, spastic aphonia, and many other dystonias including cervical dystonia, hemicrania (migraine), and some urinary disorders [9–16]. For these treatments, PTXs are mainly used because they are easily obtained and are more stable than NTXs. While these treatments are very effective, they can present serious problems in some patients in that anti-PTX, including anti-NTX, antibodies are sometimes produced after several injections. We think that the use of NTX alone may be better than using PTX because both HA1 and HA3b of PTX have immunoenhancing activity, although there are no definitive data for this immunoreactivity in humans [17]. Therefore, we established simple procedures for purifying type A NTX and for long-term storage of type A NTX at −30°C without reductions in toxicity.

**Figure 2:**
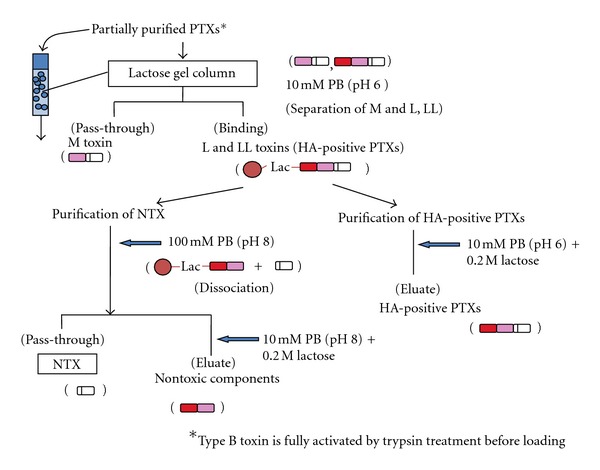
Simple procedure for purifying type A and B NTXs. Partially purified type A HA-positive PTXs (L and LL) or trypsin-treated (fully activated) type B L toxin was layered on a lactose gel column under acidic conditions (pH 6.0). After washing, the pH was adjusted to 8.0 to obtain the NTX. The bound HA-positive PTX(s) and nontoxic components were eluted with buffer supplemented with 0.2 M lactose.

 As shown in Figure 1, the HA-positive PTXs bind to galactose via HA1 under acidic conditions, and the conjugation of NTX and nontoxic components dissociates under alkaline conditions. Therefore, to isolate only the HA-positive PTXs, partially purified PTXs were first subjected to an aminophenyl beta-lactose gel column equilibrated with 10 mM sodium phosphate buffer (pH 6.0). From this column, purified preparations of HA-positive PTXs, NTX, or HA plus NTNH could be obtained by washing the column with different buffers (Figure 2). This procedure is simpler than previous protocols that used several different columns and conditions. Moreover, the NTX preparation purified by a lactose gel column showed the same toxicity per mg protein and SDS-PAGE banding profile as those obtained with previous procedures. We also found that NTX toxicity can be fully maintained for at least one year when albumin (0.05%), trehalose (1%), and Tween-20 (0.01%) were added to the storage solution [18]. The NTX, albumin, and trehalose were diluted in endotoxin-free isotonic sodium chloride solution. Finally, this purified type A NTX preparation was used as a therapeutic agent for urinary incontinence and prostate hyperplasia and as an analgesic for trigeminal neuralgia in a rat model.

 Prior to clinical use, the safety and quality of the NTX preparation and albumin were checked using NTX concentrations that were 100 folds greater than those used in treatment and albumin at 50 mg/mL. Cytotoxicity was evaluated using IMR-32 (human, abdominal), Neuro-2a (mouse, spinal cord), U937 (human, pleural effusion), NOMO-1 (human, peripheral blood), HT-29 (human, colon), Caco-2 (human, colon), HeLa (human, cervix), and Vero (African green monkey, kidney) cell lines. A mutagenicity assay of NTX and albumin was performed by an Ames test according to the preincubation technique of Yahagi et al. [19]. Prion and endotoxin contamination was assayed with a Prionics-Check Western kit (Prionics AG) and a Limulus amoebocyte lysate assay (BioWhittaker), respectively, and that of AIDS and hepatitis viruses was assayed by PCR by a commercial company (SRL, Tachikawa, Japan). In addition, approval from the ethics committees of both Okayama University and Kawasaki Medical School for this clinical trial and patient informed consent were obtained.

## 3. Clinical Trials

### 3.1. Refractory Urgency Incontinence

A preliminary study by Schurch and colleagues on 31 patients with neurogenic detrusor overactivity (NDO) demonstrated a significant increase in the mean maximum bladder capacity (296 to 480 mL, *P* < 0.016) and a significant decrease in mean maximum detrusor voiding pressure (65 to 35 cmH_2_O, *P* < 0.016) in patients injected with Botox (the main toxin used was likely LL) [14]. Seventeen of 19 patients were completely continent and very satisfied with the procedure. Interestingly, baseline improvement of urodynamic parameters and incontinence persisted in 11 patients after a 36-week follow-up period. Cruz and associates recently reported the results of a multicenter, international, randomized, double-blind, and placebo-controlled phase III clinical trials of Botox for NDO patients. Patients received 30 intradetrusor injections of either 200 U (*n* = 92) or 300 U (*n* = 91) Botox, or placebo (*n* = 92). By week 6, either 200 U or 300 U Botox significantly reduced the number of incontinence episodes per week (−21.8 and −19.4, resp.), significantly increased maximal cystometric capacity, and significantly improved quality of life scores compared to placebo. The median time to patient request for retreatment was the same for both Botox doses (42.1 weeks) [20].

Intradetrusor Botox injections have extended beyond the realm of neurogenic bladders to patients with nonneurogenic voiding and storage disorders [14, 15]. Dmochowski and associates obtained favorable effects with intradetrusor Botox injections in a double-blind, placebo controlled, randomized study that evaluated several Botox doses (i.e., 50, 100, 150, 200, and 300 U) versus placebo in 313 patients with idiopathic detrusor overactivity (IDO) [16]. Patients who experienced urinary urge incontinence with at least eight episodes/week and eight or more micturitions/day were included in the study. The primary endpoint was weekly urinary urgency incontinence episodes at 12 weeks. The authors observed a significant difference in the number of urgency incontinence episodes/week at many time points for Botox -treated patients compared to placebo patients.

 Our group performed a clinical trial using NTX to treat patients with urgency incontinence caused by NDO or by IDO. A total of 200 U (for NDO), 100 U (IDO for women), and 50 U (IDO for men) NTX were injected into the bladder wall in a minimally invasive outpatient technique using flexible cystoscopy. All patients with NDO performed clean intermittent catheterization before the treatment. Urodynamic maximum cystometric capacity and maximum detrusor pressure were evaluated before and one month after treatment by filling cystometry. Subjective and objective measures included frequency of voids and number of incontinence episodes per 24 hours from a 3-day bladder diary before treatment and 1, 3, and 6 months after treatment. A total of 16 patients with spinal NDO and a mean age of 42.3 years (range 20 to 75 years, 2 females and 14 males) and 33 with IDO and a mean age of 71.9 years (range 50 to 83 years, 15 females and 18 males) were treated [18]. NTX treatment produced an increase in the mean maximal cystometric capacity from 138.2 ± 13.8 mL at baseline to 358.8 ± 25.2 mL at one month (*P* = 0.0036) in NDO patients and 171.8 ± 10.2 mL at baseline to 319.8 ± 19.4 mL at one month (*P* < 0.0001) in IDO patients. The mean maximal detrusor pressure in detrusor overactivity decreased from 65.9 ± 6.5 cmH_2_O at baseline to 27.3 ± 4.8 cmH_2_O (*P* = 0.0025) one month after treatment in NDO patients and 63.5 ± 5.0 cmH_2_O at baseline to 32.3 ± 4.4 cmH_2_O (*P* = 0.0001) at one month for IDO patients. The mean number of daily urinary incontinence episodes decreased from 4.67 ± 0.7 to 1.07 ± 1.0 (*P* < 0.0001) at 1 month and to 2.07 ± 0.4 (*P* = 0.0002) at 3 months for NDO patients. The mean number of episodes of urinary incontinence daily decreased from 4.46 ± 0.5 to 1.49 ± 0.4 (*P* < 0.0001) at 1 month, to 1.82 ± 0.4 (*P* = 0.0002) at 3 months, and to 2.28 ± 0.7 (*P* = 0.0051) at 6 months for IDO patients. Ten of 16 NDO patients (62.5%) and 15 of 33 IND patients (45.5%) obtained complete continence within 1 month of NTX injection. NTX treatment halved the number of urinary incontinence episodes for thirteen NDO patients (81%) and 23 (69.7%) IDO patients. The mean duration of efficacy was 3 to 10 months (median 4 months) in NDO patients and 4 to 12 months (median 6 months) in IDO patients. Ten of 33 (30.3%) IDO patients had no subjective improvement. Although 5 patients had improved cystometric findings, they did not have subjective improvement due to the increase of residual urine volume and voiding dysfunction. Nineteen (57.5%) of 33 IDO patients had residual urine volumes >100 mL at 1 month posttreatment and 4 male patients required clean intermittent catheterization for a few weeks. No serious side effects occurred (Table 1).

 In our study, NTX injection therapy achieved excellent results within one month of injection. Of the 49 patients analyzed, 36 patients reported a decrease or absence of incontinence. Followup analysis of these patients showed that these effects lasted 3 to 12 months after the single treatment. While the optimum injection dose has not yet been determined, in our study, 50–100 U for IDO and 200 U for NDO were supposed to be suitable treatment doses. These results demonstrated that NTX has almost the same activity as Botox.

### 3.2. Benign Prostatic Hyperplasia

 The first off-label use of Botox to treat benign prostatic hyperplasia (BPH) in humans was reported by Maria et al. in 2003 [21]. In a randomized, placebo-controlled study, 30 men with symptomatic BPH were randomized to receive either saline or 200 U Botox, which produced clinical improvement within 1 month of treatment. By 2 months, 13 patients in the treatment group (87%) versus 3 patients in the control group (10%) reported subjective relief of BPH symptoms (*P* = 0.00001). At 12 months, the International Prostate Symptom Score decreased by 62% for the Botox-treated group, maximum urinary flow rate increased by 85%, postvoid residual urine volume decreased by 85%, and prostatic volume decreased by 61%. The prostatic-specific antigen values were also reduced by 38%. No urinary incontinence or systemic side effects were reported. In other reports of Botox treatment for prostatic hyperplasia, the increase in maximum urinary flow rate (40–121%) and the decrease in International Prostate Symptom Score (48–65.5%) were statistically significant [11–13]. There was a statistically significant reduction in prostatic volume that varied from 13.3% to 68%.

 We treated 10 male patients (mean age 70.0 years, range 61–79 years) with unsatisfactory response to alpha1-adrenoceptor blockers with 200 U (prostate volume > 30 mL) or 100 U (prostate volume < 30 mL) NTX injected into the prostate using a minimally invasive outpatient technique. Evaluation included uroflowmetry, postvoid residual urine volume, prostate volume, and International Prostate Symptom Score and was carried out at baseline and 1, 3, 6, and 12 months posttreatment. Prostate-specific antigen was measured at baseline, 6 months, and 12 months after injection. Seven out of 10 patients noted improvement within 1 month. The mean International Prostate Symptom Score decreased from 23.8 ± 7.0 to 16.3 ± 10.3 (*P* = 0.0093) at 1 month, to 14.9 ± 8.2 (*P* = 0.0074) at 3 months, and to 16.9 ± 7.3 (*P* = 0.018) at 12 months after injection. The mean prostate volume decreased from 47.8 ± 21.2 to 39.2 ± 19.5 mL (*P* = 0.0076) at 3 months.Postvoid residual urine volume improved at 3 and 6 months posttreatment. The mean prostate-specific antigen did not change during the observation period (Table 2).

 Although our study achieved only an 18% reduction in prostate volume, this outcome was similar to that obtained by Kuo and Chuang et al. [11, 12]. Botox effects appeared during the first week to one month after treatment and these benefits were maintained for 6 to 12 months [11–13]. In our study, the beneficial results were evident within 1 week of treatment and continued for 12 months. The maximum effects were from 3 to 9 months and the prostatic volume reached a minimum level at 3 to 6 months. Silva et al. [13] also reported that the prostate volume decreased gradually to a minimum level at 6 months. Therapeutic efficacy relates to the injection dosage, diffusion of toxin within the prostate, the ratio of epithelium and stromal components, and detrusor function. The improvements in International Prostate Symptom Score, maximum urinary flow rate, reduction of prostate volume, and duration of effect seen for our cases were almost the same as previous reports for Botox [11–13].

## 4. Application of NTX in a Rat Model of Trigeminal Neuropathy

Trigeminal neuropathic pain is characterized by recurrent episodes of intense, lancinating facial pain [22, 23]. Management of trigeminal neuropathic pain remains a major therapeutic challenge with antiepileptic drugs currently being the main treatment choice [24, 25]. However, since some patients cannot tolerate these drugs because of side effects (e.g., dizziness, drowsiness), more effective and safer drugs are required to treat trigeminal neuropathic pain. Botulinum toxin is one such candidate, and open-label trials with trigeminal neuralgia patients showed botulinum toxin efficacy [26, 27]. Animal studies reported that botulinum toxin injection into the peripheral tissue decreases leg neuropathic pain induced by sciatic nerve ligation or transduction [28–30]. However, a systematic review indicated that there is a lack of sufficient data that would suitably recommend botulinum toxin as an evidence-based treatment for secondary headaches or cranial neuralgias [31]. Most of these clinical trials used commercially available botulinum PTXs and did not use NTX. Since the true effects of NTX are currently unclear, continued studies on the effect of NTX treatment for trigeminal neuropathic pain animal models are needed. Here, we evaluated the effect of type A NTX in trigeminal neuropathic pain animal models.

**Figure 3:**
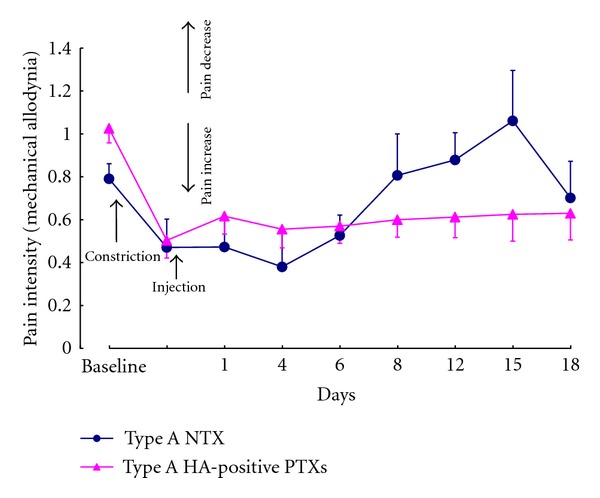
Mechanical allodynia after injection with type A NTX or PTXs in a rat hindpaw neuropathy model. Mechanical allodynia was tested with an electric von Frey filament and the hindpaw withdrawal thresholds measured. The data were divided by the contralateral naïve hindpaw data. After sciatic nerve contraction, the ipsilateral hindpaw threshold was decreased. Ipsilateral injection with NTX or HA-positive PTXs produced the same toxicity (10 U/0.1 mL) in mechanical allodynia. The effects of NTX on decreasing pain levels were longer lasting than HA-positive PTXs. *n* = 4; data represent the mean ± SEM.

 Kitamura et al. [32] reported that a large decrease in head withdrawal threshold was observed after ipsilateral infraorbital nerve (branch of trigeminal nerve) constriction (IoNC) by stimulating the face with an electric von Frey filament. This result showed that the rats had tactile allodynia, which trigeminal neuropathic pain patients usually show clinically and which is considered to be a pain response. The intradermal ipsilateral injection of type A NTX (100 pg or 10 U) significantly increased the head withdrawal threshold compared to saline-injected trigeminal neuropathic pain rats, suggesting that NTX injection decreased rat trigeminal neuropathic pain. These results are consistent with those of Filipović et al. [33], who also found thatunilateral injection of Botox reduced IoNC-induced allodynia with the effect lasting more than 2 weeks. Next, we compared the analgesic effect of type A NTX and HA-positive PTXs (a mixture of L and LL toxins obtained using a lactose gel column as shown in Figure 2).  Figure 3 shows that, compared to PTX, NTX decreased pain levels for longer periods.

 Kumada et al. [34] tested freely moving rats and found that type A NTX attenuated IoNC-induced thermal hyperalgesia. These effects were dose dependent (2–200 pg) and statistically significant at 100 and 200 pg (*P* < 0.05). Off-site (neck) injection of type A NTX did not relieve thermal hyperalgesia and coinjection of type A NTX with a neutralizing antibody in the area of infraorbital nerve innervation prevented relief of thermal hyperalgesia. These results strongly suggest that intradermal injection of type A NTX in the area of infraorbital nerve innervation alleviates IoNC-induced thermal hyperalgesia.

 The detailed mechanisms of the type A NTX effects on trigeminal neuropathic pain in animal models are not clear. Some studies suggest that the botulinum toxin undergoes axonal transport. Antonucci et al. [35] reported that, after injection of type A NTX into rat whisker muscles, cleaved synaptosomal-associated protein of 25 kDa (SNAP-25) appeared in the facial nucleus, indicating that the toxin migrated along the axons and underwent neuronal transcytosis. Matak et al. [36] found that Botox truncated SNAP-25 in the medullary dorsal horn (spinal trigeminal nucleus) was evident 3 days following the peripheral treatment. Filipović et al. [33] reported that unilateral Botox injection reduced-IoNC induced bilateral dural extravasation while Kitamura et al. [32] observed that neurons isolated from trigeminal ganglion ipsilateral to infraorbital nerve constriction exhibited significantly faster onset of FM4-64 dye release in dissociated trigeminal ganglion neurons compared to neurons in contralateral sham surgery. Intradermal injection of type A NTX in the area of infraorbital nerve innervation reduced the exaggerated FM4-64 dye release in trigeminal ganglion neurons from these rats.

 In this manuscript, we demonstrated that type A NTX purified with a lactose gel column could be used clinically and was as effective as commercially available PTXs. Recently, preparations of type A NTX alone and type B PTX (probably L toxin) have been made commercially available. The production of anti-NTX Ab in repeatedly injected patients as well as the efficacy of the toxin must be monitored. Usually, type B toxin is not fully activated but can be activated following trypsin treatment. Our procedure is suitable for purifying fully activated type B toxin with partially purified type B PTXs first fully activated by trypsin just prior to loading on the lactose gel column. After binding of the L toxin to the column, trypsin was washed out and the fully activated L toxin or NTX can then be obtained similarly to type A toxins (Figure 2) [37]. We also found that the toxicity of type B NTX could be maintained at −30°C for long periods by adding 0.05% albumin. We plan to evaluate fully activated type B NTX for clinical usage in the future.

## Figures and Tables

**Table 1:** Change of parameters before and after intradetrusor NTX treatment of NDO and IDO patients.

		Baseline	1 month	3 months	6 months
NDO	MCC (mL)	138.2 ± 13.8	358.8 ± 25.2*	—	—
	Pdetmax (cmH_2_O)	65.9 ± 6.5*	27.3 ± 4.8*	—	—
	Frequency	7.53 ± 0.7*	5.71 ± 0.3*	6.31 ± 0.4*	—
	Incontinence (times/day)	4.67 ± 0.7*	1.07 ± 1.0*	2.07 ± 0.4*	—

IDO	MCC (mL)	171.8 ± 10.2	319.8 ± 19.4*	—	—
	Pdetmax (cmH_2_O)	63.5 ± 5.0*	32.3 ± 4.4*	—	—
	Frequency	13.7 ± 0.6*	11.4 ± 0.7*	11.2 ± 0.7*	10.5 ± 0.9*
	Incontinence (times/day)	4.46 ± 0.5*	1.49 ± 0.4*	1.82 ± 0.4*	2.28 ± 0.7*

*Compared with baseline, *P* < 0.05.

MCC: maximum cystometric capacity; Pdetmax: maximum detrusor pressure at detrusor overactivity.

**Table 2:** BPH Patient profiles and results after intraprostatic NTX treatment.

	Baseline	1 week	1 month	3 months	6 months	9 months	12 months
Number of patients	10	10	10	10	9	9	8
IPSS	23.8 ± 7.0	19.4 ± 9.3*	16.3 ± 10.3*	14.9 ± 8.2*	13.8 ± 7.5*	13.8 ± 7.6*	16.9 ± 7.3*
Storage	8.7 ± 4.8	7.2 ± 4.5	6.7 ± 5.4	5.3 ± 3.8*	4.4 ± 3.5*	5.4 ± 3.5*	5.2 ± 4.3*
Voiding	11.8 ± 3.4	9.5 ± 4.4	6.9 ± 5.0*	7.4 ± 4.8*	7.0 ± 4.2*	6.8 ± 4.5*	7.4 ± 4.6*
QOL score	5.2 ± 1.0	4.3 ± 1.6*	3.4 ± 1.6*	3.3 ± 1.8*	3.2 ± 1.6*	3.7 ± 1.4*	4.3 ± 1.5*
Qmax, mL/s	6.3 ± 3.1	5.5 ± 2.0	6.1 ± 2.6	8.8 ± 2.9*	6.8 ± 3.9	6.2 ± 2.2	7.2 ± 4.0
Residual urine (mL)	99.5 ± 94.5	84.9 ± 83.1	101.5 ± 97.0	65.3 ± 73.6*	52.9 ± 62.5*	86.3 ± 100	72.4 ± 58.2
Prostatic volume (mL)	47.8 ± 21.2	45.9 ± 22.3	40.2 ± 18.2*	39.2 ± 19.5*	40.2 ± 19.2*	42.9 ± 23.2	41.0 ± 17.0
PSA (ng/mL)	4.30 ± 3.0				3.75 ± 2.2		4.35 ± 2.8

*Compared with baseline, *P* < 0.05.

## Abbreviations

NTX: Neurotoxin
PTX: Progenitor toxin
HA: Hemagglutinin
NTNH: Nontoxic non-HA
NDO: Neurogenic detrusor overactivity
IDO: Idiopathic detrusor overactivity
IoNC: Infraorbital nerve constriction.
